# Extract antibody and antigen names from biomedical literature

**DOI:** 10.1186/s12859-022-04993-4

**Published:** 2022-12-06

**Authors:** Thuy Trang Dinh, Trang Phuong Vo-Chanh, Chau Nguyen, Viet Quoc Huynh, Nam Vo, Hoang Duc Nguyen

**Affiliations:** 1 Center for Bioscience and Biotechnology, University of Science, Ho Chi Minh City, Vietnam; 2 Vietnam National University, Ho Chi Minh City, Vietnam; 3 Laboratory of Molecular Biotechnology, University of Science, Ho Chi Minh City, Vietnam

**Keywords:** Antibody, Antigen, Corpus, Named entity recognition, BioNLP, Semi-automatic annotation, Deep learning, ABAG-NER tool

## Abstract

**Background:**

The roles of antibody and antigen are indispensable in targeted diagnosis, therapy, and biomedical discovery. On top of that, massive numbers of new scientific articles about antibodies and/or antigens are published each year, which is a precious knowledge resource but has yet been exploited to its full potential. We, therefore, aim to develop a biomedical natural language processing tool that can automatically identify antibody and antigen entities from articles.

**Results:**

We first annotated an antibody-antigen corpus including 3210 relevant PubMed abstracts using a semi-automatic approach. The Inter-Annotator Agreement score of 3 annotators ranges from 91.46 to 94.31%, indicating that the annotations are consistent and the corpus is reliable. We then used the corpus to develop and optimize BiLSTM-CRF-based and BioBERT-based models. The models achieved overall F1 scores of 62.49% and 81.44%, respectively, which showed potential for newly studied entities. The two models served as foundation for development of a named entity recognition (NER) tool that automatically recognizes antibody and antigen names from biomedical literature.

**Conclusions:**

Our antibody-antigen NER models enable users to automatically extract antibody and antigen names from scientific articles without manually scanning through vast amounts of data and information in the literature. The output of NER can be used to automatically populate antibody-antigen databases, support antibody validation, and facilitate researchers with the most appropriate antibodies of interest. The packaged NER model is available at https://github.com/TrangDinh44/ABAG_BioBERT.git.

## Background

Antibodies (ABs), also referred to as immunoglobulin, are host proteins secreted by plasma cells to serve as the first response against targeted antigens (AGs), which are foreign molecules or organisms that the ABs stringently bind to and ultimately neutralize in various ways. The ability of ABs to bind AGs with a high degree of affinity and specificity has led to their ubiquitous use in a variety of scientific and medical disciplines: diagnoses, therapeutics, analysis, purification, enrichment, mediation, and modulation of physiological responses [1].

Owing to their profound impact on human’s healthcare, a vast array of scientific discoveries regarding ABs and their AGs have been introduced each year. As of June 2021, there were over 2 million research articles about antibody and/or antigen (ABAG) on NCBI PubMed. This is undoubtedly an enormous source of knowledge about ABAG essential for further research, diagnostic, and therapeutic purposes. Unfortunately, such an important source of knowledge has not yet been exploited effectively.

In an effort to facilitate the process of AB search and validation through such “big data”, numerous projects have emerged over the past decade. For example, antibody databases like Antibody Exchange [2], Antibody Watch [3], SAbDab [4], Antibody Registry [5], etc. have been collecting, cross-referencing, and unifying a variety of information about ABs and the supporting evidence. Among existing antibody databases, AntiBodies Chemically Defined (ABCD) database [6] sufficiently covers general information about antibodies and their targets that are corroborated by PubMed articles or patents. Despite being an extensive resource, as a manually curated depository, ABCD (version 9.0, updated in August 2020) had only 3231 PubMed IDs (PMIDs), which evidently did not cover all over 2 million PubMed articles related to ABAG. Additionally, authors usually only deposit ABs and AGs that are the main topics of their published articles. Hence, not all ABAG mentioned in articles are listed in the database.

On that account, together with the constantly growing volume of publications on ABAG topics, there exists a high demand for a platform that can automatically collect, process, and extract key information about antibodies and antigens from relevant biomedical texts. One of the most potent solutions, BioNER is a task of recognizing predefined biomedical-related entities: chemicals, genes/proteins, diseases, or antibodies and antigens, in our case, that are mentioned in massive and unstructured biomedical texts. BioNER, and NER in general, plays an essential role as a foundation for many downstream applications such as knowledge base construction, relation extraction, question answering, and other text mining tasks [7]. Traditional NER techniques that utilize unsupervised learning typically demand an exhaustive lexicon and are hard to transfer to other domains. In a superior approach, deep learning is advantageous in automatically finding hidden features [7]. Composed of multiple processing layers, typically artificial neural networks, deep learning models can learn multi-level representations of complex and intricate features from data via non-linear activation functions. More importantly, since the learning of features and useful representations is automatic and directly from raw data, without the need for manually designed features, deep learning models are not only effort-saving but also domain-independent [8]. Examples of deep learning neural networks for sequential data include Convolutional Neural Network (CNN)- or Recurrent Neural Network (RNN)-based models in NER, especially in domain-specific BioNER [9, 10].

Notably, Long-Short Term Memory (LSTM), a special case of RNN, has superiority in remembering larger-context information with its gate mechanism that decides to forget irrelevant information and only allows important information to pass through. This release of unhelpful memories efficiently averts memory explosion. In addition, Bidirectional Long-Short Term Memory—Conditional Random Field (BiLSTM-CRF), introduced by Huang et al. [11] and by Lample et al. [12], has been one of the most widely used models in sequence labeling tasks, including BioNER. BiLSTM involves two LSTM networks, one reads sequences from left to right (forward) and one from right to left (backward). While the forward operation handles information from the past, the backward layer is for information from the future and hence overall, BiLSTM covers a wider context that is useful for the predicting task [13]. Next, the output from BiLSTM, which is a rich contextual vector representation of the input sequence, is passed to a CRF (Conditional Random Field) layer. Using a probabilistic sequence-labeling model for sequence tagging, CRF not only combines the context information from BiLSTM outputs, but also considers dependencies and strong restrictions between the output sequence of labels for its prediction. Character-level embeddings can also be included into BiLSTM-CRF to enhance model performance as they help deal with out-of-vocabulary or misspelled words, or entity mentions of multi-form, etc. [13].

Recent advancements in NER also take advantage of Bidirectional Encoder Representations from Transformers (BERT). Introduced by Lee et al. [14], BioBERT is a biomedical domain-specific language representation model. BioBERT was initialized with the weights from the pretrained Google BERT model and further pretrained on large-scale PubMed abstracts and PubMed Central full-text articles for biomedical task-specific labeling [14]. With a masked language model architecture to learn bidirectional representations, BioBERT excels in representing words in complicated contexts like biomedical literature [15, 16]. BioBERT also outperforms other models when it comes to polysemous words, as it produces different embeddings for different meanings of the same word [17]. Subsequently, BioBERT has been applied to improve BioNER performance, especially at determining the correct name boundaries of biomedical entities such as disease, drug/chemical, gene/protein [14]. Using BioBERT as word embeddings can also generate contextualized representations of complex biomedical texts, facilitating downstream tasks [14, 17]. For example, Gondane utilized BioBERT as feature embeddings for inputs to a dense fully connected neural network that identifies personal health experience mentioned in tweets [18].

To effectively train a deep learning model, a well annotated dataset is essential. However, to the best of our knowledge at the time of our study, there has been no corpus for antibody and antigen entities. There have been several corpora in closely related domains. For example, PGxCorpus [19] covers chemical, gene/protein, disease, phenotypes, haplotype, and gene variations. The construction of this corpus followed a semi-automatic annotation process, with automatic pre-annotation and manual correction, which was also the approach for our corpus annotation. The Inter-Annotator Agreement (IAA) strict F1 for this corpus was 57.4%. JNLPBA [20] is a well-known corpus for protein and gene entities. It contains 2404 abstracts and has been used as a benchmark corpus in a lot of state-of-the-art (SOTA) NER studies [21]. The corpus contained 25 k sentences, 569 k tokens, and 35 k annotations in total. Another similar corpus is ProGene [22], which was developed more recently (in 2020 by Faessler et al.) and of a slightly larger scale with 3308 abstracts. This gene-protein corpus was further grouped into 5 specific entities: protein molecule, protein family/group, protein complex, protein variant, and elliptic enumeration of protein. Despite several variants for protein corpora, no work has been done for antibodies or antigens.

The ultimate aim of this study is to develop an automatic tool for identifying antibody-antigen names in large-scale biomedical abstracts. Our ABAG-NER tool would potentially extract data from the scientific literature to populate a database for antibodies and antigens. To that end, the two main objectives in our study are (1) to construct an annotated corpus for antibody and antigen entities and (2) to build a BioNER model for antibody and antigen name recognition.

## Methodology

### Data collection

The very first step in our procedure is to obtain essential information, including AB names and synonyms, AG names and synonyms, and PMIDs from the ABCD database. The PMIDs are used to further retrieve abstract texts from PubMed, while all the names and synonyms are used to build a label lexicon.

We further manipulated a great number of AB names in ABCD with the pattern of “anti-AG_Name-AB_Name” or similar (e.g., anti-HER2-2Rs15d), and extracted more AB and AG names (2Rs15d and HER2, respectively) to add to the label lexicon. We also filtered out the lexicon names that are easily misleading:

- Single-letter names such as “E”, “G”, and “S”, which are abbreviations of envelope-, lycol-, and spike-proteins, respectively
- Names that are less-than-1000 numbers (easily mistaken with numbers in measurements)
- Names that are common words like “antigen”, “antibody”, “fab”, “mab”, “vhh”, “mg”, …

### Corpus construction using semi-automatic annotation

#### Automatic annotation tool

**Fig. 1:**
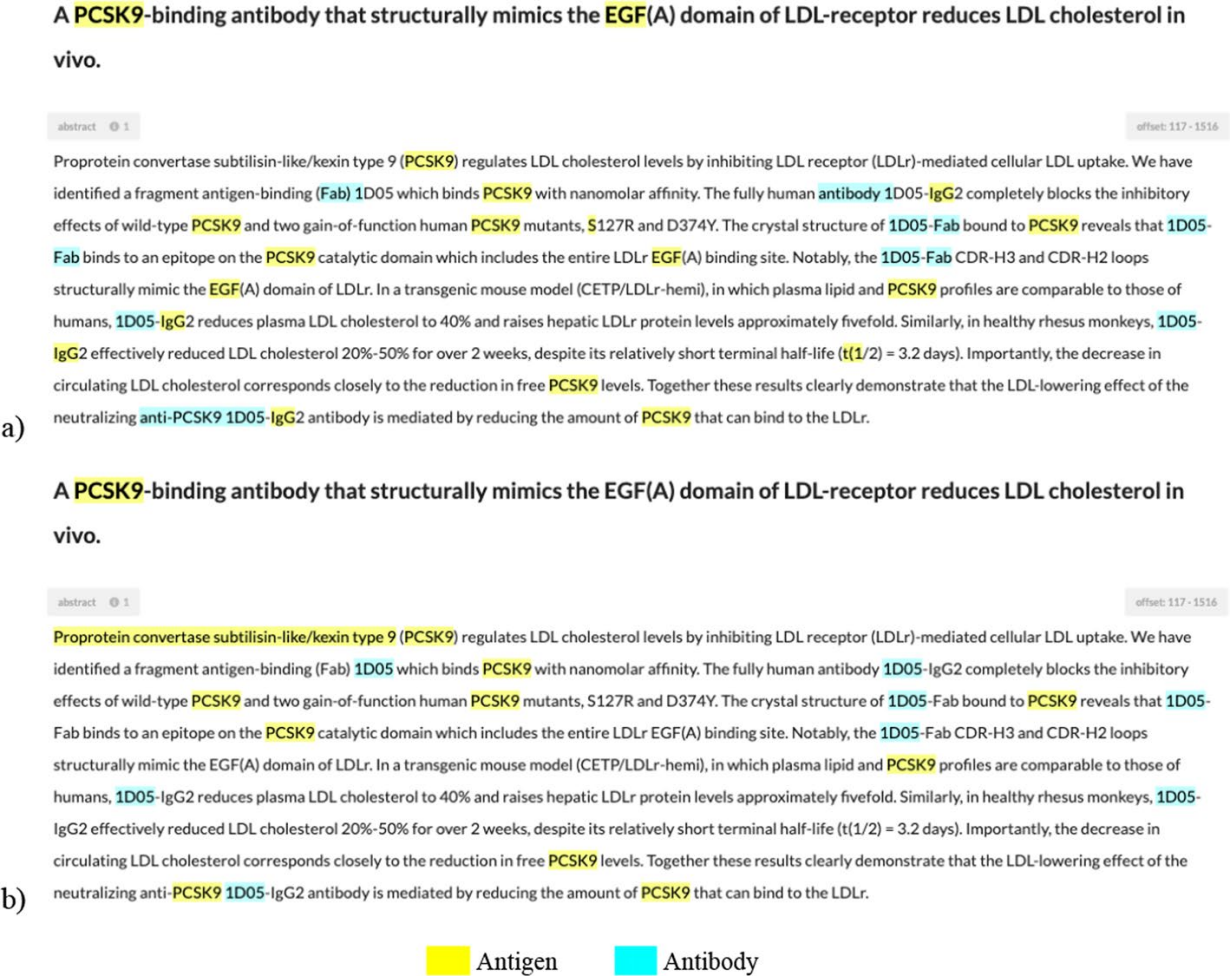
An example of annotation with ezTag [before (**a**) and after (**b**) manual annotation]. Yellow highlight is AG; blue highlight is AB

We utilized a web-based concept tagging tool named ezTag [23] during our annotation tasks, both in automatic and manual phases. Since ezTag uses a string-match algorithm for automatic annotation, we provided to ezTag the label lexicon and 3210 PubMed abstracts as inputs. The outputs were automatically annotated abstract texts with tagged entities in highlight as illustrated in Fig. 1a.

#### Manual revision_annotation guidelines

The automatically annotated abstract texts contained significant numbers of errors (further illustrated in Results and Discussion), which unequivocally required manual revision by human annotators.

Three annotators, trained intensively for the annotation tasks, each independently reviewed and corrected 1070 abstracts, following stringent criteria for consistency in annotation text span, entity type, and coverage. Some of the top prioritized guidelines included:

1. If a mention is in the format of “anti-” + AG_name + “Antibody”/“mAb”/…, we annotate the AG_name as “Antigen”; we do not annotate the whole phrase as "Antibody"
2. Annotate names only, exclude common words like “antibody”, “antigen”, “protein”, “mAb”, “Fab”, “scFv”, “VHH”, … that come before or after the name, unless these words are part of the name
3. Include species names mentioned along with AB/AG names, if they are mentioned in either of the two following formats:
  - Species_name + AB/AG_name
  - AB/AG_name + “of” + Species_name (annotate the whole phrase as a single entity)
4. Do NOT annotate antibody type (IgG, IgM, IgA, IgE, IgD, …), region, loop, fragment name of antibody, or domain name of target, unless they are the main topic/target of the AB-AG interaction in the abstract

All the guidelines and rules were established before the beginning of manual annotation tasks and regularly refined at weekly meetings for consistent annotations. The entire annotation process took approximately 1.5 months.

### Inter-annotator agreement

To ensure the corpus reliability, we performed Inter-Annotator Agreement (IAA) statistics via TeamTat, an annotation webtool supporting multi-annotator collaborative work [24]. We performed double annotation for 10% (321 abstracts) of the corpus and triple annotation for 1% (32 abstracts). All doubly- and triply-annotated abstracts were chosen randomly to ensure the population representation. IAA score indicates consistency among annotators in tagging named entities and was measured using entity-level F1 measures:

- (TP: True Positive; FP: False Positive; FN: False Negative).
- Precision (P):P = TP/(TP + FP), which represents positive predictive value, or relevancy of retrieved values.
- Recall (R):R = TP/(TP + FN), which represents sensitivity, or retrieval of relevant values.
- F1: F1 = 2*P*R/(P + R), the balanced ratio between P and R.

F1 has been commonly used in NER tasks [14]. For all our results, we reported micro F1. Evaluation of either IAA or NER models in our study used both exact-match (a predicted annotation by model and its ground-truth annotation from the corpus must match exactly, both the text boundary and the entity type, to be considered correct) and relax-match F1 scores. Although exact-match evaluation is widely used, it is unable to judiciously assess bioNER performance. This is because biomedical entities are frequently written in various forms in biomedical texts. For example, “(6–4)photoproduct”, “6,4-photoproduct”, and “(6–4) Photoproduct” all refer to the same entity. The venial mismatch in just a hyphen, space or brackets obviously makes no difference in meaning, but exact-match marks it incorrect, which costs the coverage and results in a low F1 score. Therefore, relax-match evaluation was introduced to account for this flexibility. Relax-match is further divided into span mismatch relax and type mismatch relax. With span mismatch relax, an annotation is counted as correct if it has the correct entity type with the ground-truth tag, regardless of their mismatched text boundaries. With type mismatch relax, a boundary-matched annotation is adequately counted as correct, regardless of its entity type [7].

### The annotated corpus

**Fig. 2:**
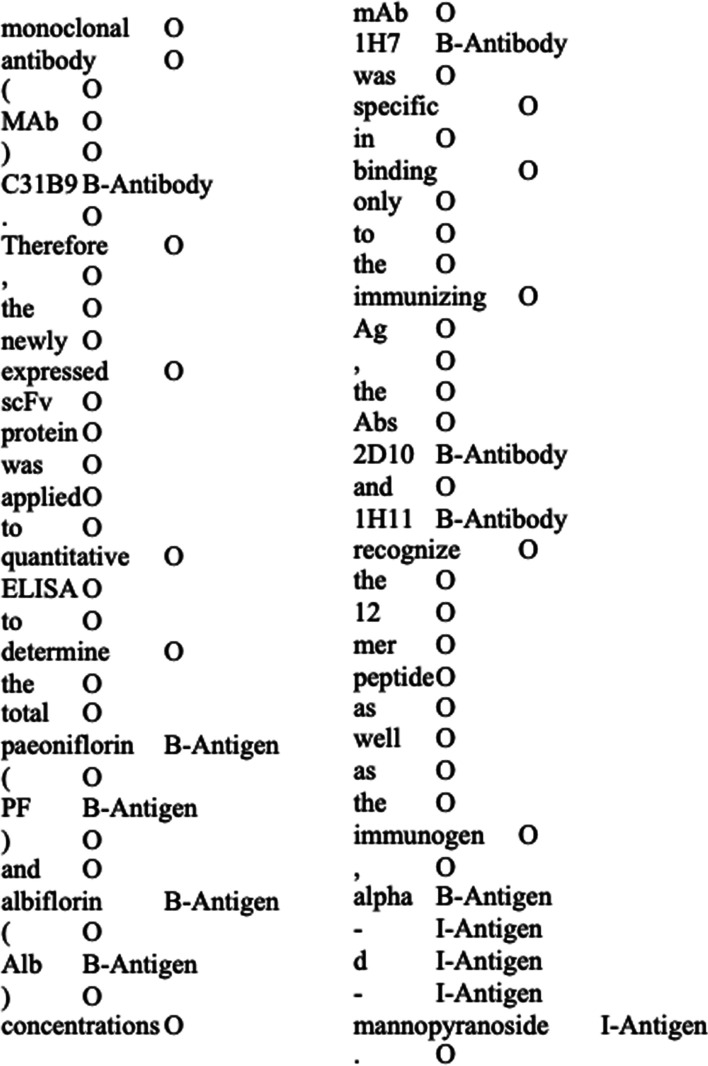
Excerpts of our ABAG annotated corpus in IOB tagging scheme

The final, fully annotated corpus was used to train and evaluate NER models. For this purpose, all the annotated abstract files were converted from XML to CoNLL tabular format where entity types followed the Begin (B), Inside (I) and Outside (O) scheme (Fig. 2). During the conversion, abstract tokenization was performed using SpaCy NLP toolkit with additional Python regular expression script to further separate the tokens by some special characters: r'[− ~ /: + ()\'\][",_. > *•#]'. In addition, all tokens were kept case sensitive.

## Model optimization

### Models

The project employs BiLSTM-CRF and BioBERT for generation of baseline NER models.

**Fig. 3:**
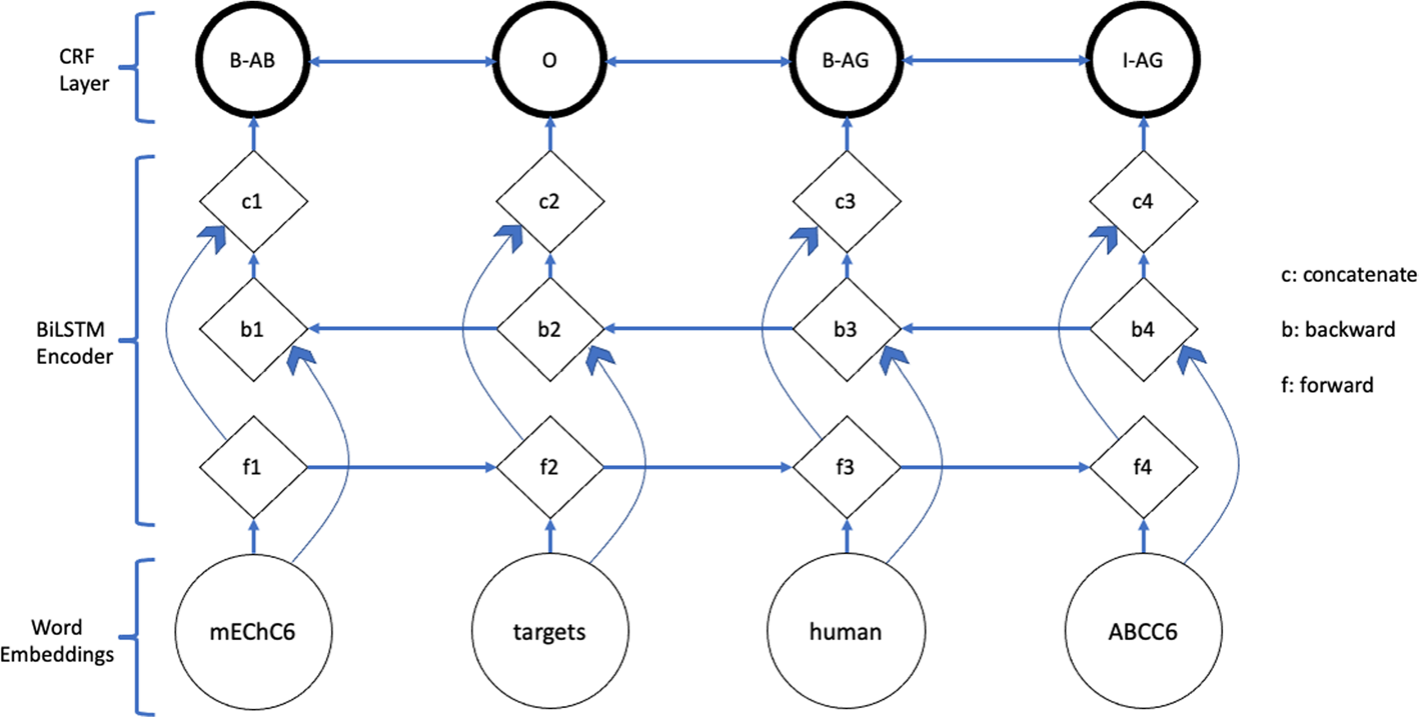
BiLSTM-CRF architecture employed in anaGo model

Firstly, BiLSTM-CRF was obtained via anaGo library; the version used in our study was anago-py367 [25], which was suitable to run with Python 3.7. Developed and optimized by Nakayama in 2017 with the combined technique BiLSTM-CRF [26], anaGo was implemented in Keras for NER and many other sequence labeling tasks. anaGo implements different pre-trained word embeddings as input; it also has the capability to self-generate word embedding based on training data [12, 27]. The BiLSTM-CRF architecture is described in Fig. 3.

The input to the model is a sequence of tokens (“mEChC6 targets human ABCC6”). The word embedding, which can be pre-trained or self-generated based on training data, encodes each token and inputs the corresponding representation to Bi-LSTM neural networks. Bi-LSTM uses two LSTM networks, forward (f1–4) and backward (b1–4). The vector representations from both networks are concatenated (c1–4) and inputted to the CRF tagging layer for label assignment [12, 13, 27].

The model consists of 10 layers with over 2 M parameters. The model hyperparameters were kept as default: dimension of words = 100, dimension of characters = 25, Adam optimizer, dropout = 0.5, and batch size = 32. The number of training epochs was set to 100 at maximum, with early stopping that usually stopped at approximately the 60th epoch; the early stopping condition was when the validation loss of the model did not decrease more than 2 × 10^–4^ (~ 0.01% initial loss) after 5 consecutive epochs. We set this loss threshold since the validation F1 typically did not increase afterward.

Secondly, we also used BioBERT, a transformer-based model initialized with BERT by Google and pretrained with 18B words from biomedical texts by Lee et al. [14]; the version used in our study was BioBERT-Base v1.1. Similar to BiLSTM-CRF training, the model hyperparameters for BioBERT fine-tuning were mostly kept as default, with AdamW optimizer and a learning rate of 4 × 10^–5^. We adjusted the maximum sequence length up to 512 and batch size down to 16. The number of training epochs was set to 5, but the model usually obtained the best performance at the 2nd or 3rd epoch.

### Data splitting

**Fig. 4:**
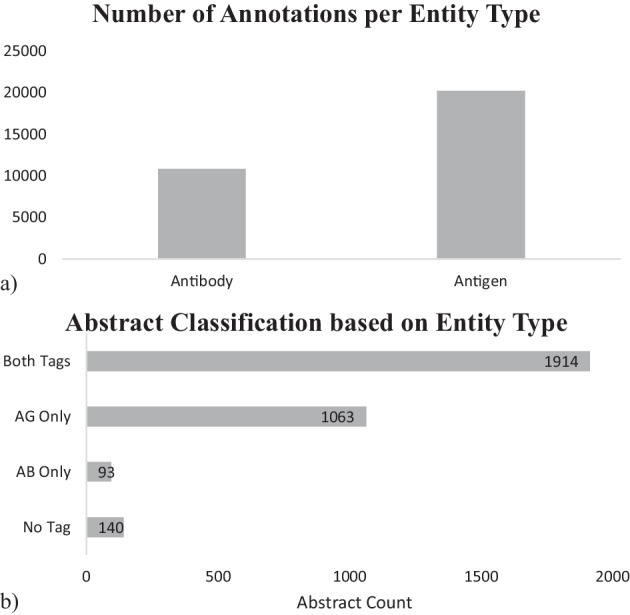
Entity type distribution in the annotated corpus. **a** Total number of AB and AG, mentioned in the corpus; **b** 4 groups of abstract classification based on presence of entity types

For optimal performance, we investigated 3 different data splitting ratios and 2 different data clustering methods. For the former, NER model training typically sets 10–20% data for testing, thus we evaluated 3 different splitting ratios of 10%, 15%, and 20% testing. The three ratios of train:validate:test including 60:20:20, 70:15:15, and 80:10:10 were assessed. Each ratio had three replicates of different random seeds. For the clustering methods, we tried randomizing data on the whole dataset (3210 abstracts) versus randomizing within each abstract classification group (4 groups listed in Fig. 4b). Due to the unequal distribution of abstracts across the 4 groups, especially with only 93 AB-only abstracts and 140 no-tag abstracts (Fig. 4b), different data clustering methods were necessary to ensure equal distribution of each type into train-validate-test sets.

### Evaluation

Both exact-match and relax-match F1 scores were computed to assess the performance of the NER models. Additionally, error analysis on the model outputs was conducted on ten randomly chosen abstracts in the test set as well as eight relevant PubMed abstracts outside the corpus; the process served as a detailed examination of model performances and major error types.

## Results

### Corpus statistics

ABCD_v9 provided total 40,127 names and synonyms (27,754 AB and 12,373 AG); pre-processing to extract more names from ABAG complex and to filter out confusing names (as described in Methodology) gave total 48,653 names (35,654 AB and 12,999 AG). These names were used to build the label lexicon inputted to ezTag for automatic pre-annotation.

ABCD_v9 provided 3233 PMIDs, of which 3210 abstracts were available through PubMed. Consequently, the final corpus contains 3210 abstracts, with average 7.5 sentences and 183 tokens per abstract. Automatic pre-annotation on these abstracts produced 38,471 tags. After manual annotation, there remained 31,061 tags in the whole corpus, of which 10,835 were AB and 20,226 were AG, as illustrated in Fig. 4a. On average, there are about 10 annotations per abstract, approximately 3.5 for AB and 6.5 for AG.

After manual revision, the number of annotations decreased by over 7000 instances, which indicated that automatic annotation based on text-matching algorithms tended to introduce false positive (FP) errors. Consequently, the manual corrections involved mostly removing non-sense mentions, which were typically abbreviations or measurements sharing common name formats with ABAG and mistakenly annotated. As illustrated in Fig. 1a, automatic annotation tagged “Fab”, “IgG”, “S”, or “t(1”, which are too generic or non-sense mentions. Manual corrections (Fig. 1b) removed these annotations. Moreover, the main corrections also consisted of adding many ABAG names being mentioned but not the main topics of the abstracts, which were not covered in ABCD, and re-tagging multi-token labels to ensure that the annotations all followed our principles. For example in Fig. 1b, the full name (non-abbreviation) of AG “Proprotein convertase subtilisin-like/kexin type 9” was manually added and the AB annotation “anti-PCSK9 1D05” was re-tagged with “PCSK9” as AG and “1D05” as AB to match our annotation rules.

As depicted in Fig. 4b, our corpus covered all four possible cases: abstracts containing both AB and AG tags (1914 abstracts), abstracts containing only AB (93 abstracts), abstracts containing only AG (1063 abstracts), and abstracts with neither AB nor AG (140 abstracts).

We observed that the corpus covered a wide range of biomedical topics, from synthetically therapeutic antibodies (anti-HIV, anti-SARS-CoV, etc.), humanization of antibodies, nanobodies, study of crystal structure, mutation analysis, target screening assays, to naturally pathogenic autoantibodies (anti-DNA, anti-collagen, etc.), and so on. This diversity assisted model training in learning to recognize entities (or ignore non-entities) from different cases and contexts.

### Inter-annotator agreement evaluation

The consistency among annotators working on this ABAG corpus was measured via Inter-Annotator Agreement (IAA) F1 scores, reported in Table 1.

**Table 1:** Inter-annotator agreement (IAA) F1-measures of the ABAG annotated corpus

FA (%)	CA (%)	PA (%)	DA (%)	SN (%)	Strict F1 (%)	Relax F1 (%)
86.54	0.20	2.46	0.03	10.77	**91.46**	**94.31**

Bold numbers are the overall F-measures, which are the main scores to assess the corpus quality

Relax-match evaluation divides the agreement into 3 levels: full agreement, partial agreement, and single annotation. Full agreement (FA) requires the 2 annotations to have the exact same entity type and text span. Partial mismatches (concept agreement, partial agreement, and disagreement) are considered as false in strict F1 but are correct in relaxed F1. Concept Agreement (CA) means that the two annotations have the same text span but are tagged as different entity types; Partial Agreement (PA) means the same entity type for overlapping text span between the 2 annotations; Disagreement (DA) means different entity types for overlapping text span. Single (SN) annotation is tagged by only some, but not all, annotators; basically, SN is the sum of False Positive and False Negative. F1 scores were calculated as:

- Strict F1 = FA/(100% − SN/2) * 100%
- Relax F1 = (FA + CA + PA + DA)/(100% − SN/2) * 100%

The overall F-measure is 91.46% for strict method and 94.31% for relax method (Table 1), which is in the ‘almost perfect agreement’ range of F = 81–100%, according to the Landis and Koch scale [28, 29].

### Dataset distribution

To optimize both the BiLSTM-CRF and BioBERT models, we surveyed two different methods of data random clustering (whole corpus versus group-wise) and three different splitting ratios (60:20:20, 70:15:15, and 80:10:10). Statistics of data in each set were performed to ensure uniform distribution.

Table 2 reported statistics of the corpus with group-wise random clustering method and splitting ratio of 60:20:20. The same average sentences and similar average mentions per abstract indicated a uniform distribution of the corpus abstracts across the training, developing, and testing sets. In addition, AB and AG annotations were also equally distributed in an approximately 6:2:2 ratio. Overall, group clustering with the ratio of 80:10:10 achieved the best performance.

**Table 2:** Statistics of the ABAG annotated corpus with 60:20:20 splitting ratio and group-wise random clustering method

Characteristics	Training	Developing	Testing	Total
No. of PubMed article abstracts	1930	640	640	3210
No. of antibody mentions	6627	1948	2260	10,835
No. of unique antibody mentions	1963	666	796	3144
No. of antigen mentions	12,198	3981	4047	20,226
No. of unique antigen mentions	3235	1335	1271	4950
Avg. sentences per abstract	7.5	7.5	7.5	7.5
Avg. words per abstract	182.9	181.8	183.5	182.8
Avg. mentions per abstract	9.8	9.3	9.9	9.7

## NER model performance

### BiLSTM-CRF

First, the BiLSTM-CRF model achieved the highest F1 score of 62.49%, with a precision score (66%) much higher than the recall score (58%) (Table 3). These scores are micro-averaged from the total true positives, false negatives, and false positives.

**Table 3:** An example BiLSTM-CRF run with 80:10:10 data splitting ratio and whole-corpus random clustering method

Entity	Precision	Recall	F1 Score	Support
Antibody	74	56	63	1164
Antigen	63	60	61	2044
Micro average	66	58	62	3208

F1 for AB was 63%, higher than F1 of 61% for AG. In addition, while precision was higher than recall for both AB and AG, recall of AB was surprisingly low (about 20% less than precision) and even lower than recall of AG (Table 3). For BiLSTM-CRF, the early stopping on average stopped at approximately 60th epoch, where no improvement for the validation set was detected, which was consistent at every run, so both the upper limit of 100 epochs and the applied early stopping conditions were reasonable.

### BioBERT

Second, the BioBERT model achieved the highest F1 of 81.44%, which was almost 20% higher than the F1 of BiLSTM-CRF for both entities.

F1 for AB was 88%, much higher than F1 = 78% for AG. Noticeably, R scores were higher than P scores for both AB and AG, with recall for AB reaching over 90% (Table 4). This trend was opposite to that of the BiLSTM-CRF model. Overall, BioBERT clearly showed improvement in annotation coverage compared to BiLSTM-CRF (over 20% higher R scores).

**Table 4:** An example BioBERT run with 70:15:15 splitting ratio and group-wise random clustering method

Entity	Precision	Recall	F1 Score	Support
Antibody	86.15	90.36	88.20	1576
Antigen	74.60	81.84	78.05	3072
Micro average	78.40	84.72	81.44	4648

#### Relax-match evaluation

Relax-match F1 was calculated to provide a fairer understanding of model performance. While type relaxation (or categorical relaxation, which does not differentiate the 2 AB and AG entity types) reached 63.8%, which increased by 2.5% compared to strict F1, span (or text boundary) relaxation reached 71.7%, which increased by 10.4% overall. The BiLSTM-CRF model achieved 75.4% (14.1% increase) if both criteria were relaxed. The improvement trends for AB and AG were parallel to each other and also similar to that of the overall (Table 5).

**Table 5:** Relax-match F1 Evaluation for BiLSTM-CRF model with 80:10:10 splitting ratio & whole-corpus random clustering method

Relax type	Strict	Type relax	Span relax	Relax both
Antibody	68.76	70.85	77.58	80.85
Antigen	56.89	59.57	68.20	72.18
Overall	61.34	63.80	71.72	75.44

Similar trend was observed for the BioBERT model but with smaller increases in F1 score (Table 6). The BioBERT model achieved 88.74% (7.77% increase) if both criteria were relaxed; the score improvement was about half of that of the BiLSTM-CRF model. This can be attributed to the high performance of BioBERT, especially in recall and type distinction, that the relax evaluation did not make as much difference.

**Table 6:** Relax-match F1 Evaluation for BioBERT model with 70:15:15 splitting ratio and group-wise random clustering method

Relax type	Strict	Type relax	Span relax	Relax both
Antibody	87.57	87.94	92.74	93.54
Antigen	77.65	78.24	85.5	86.33
Overall	80.97	81.48	87.92	88.74

## Discussion

Many antibody databases focus mostly on structure, sequence, specificity, and source of antibodies. At the time of our research, ABCD was the only one with all general information required for our semi-automatic annotation project (names and synonyms of antibodies and their targets, together with the PubMed IDs of articles supporting the information) being systematically formatted and readily available. Through PMIDs obtained from ABCD, PubMed abstracts were directly retrieved via ezTag. Because ezTag supports both approaches: automatic annotation and manual annotation [23], we could continue manual editing directly on the results of automatic annotation. Moreover, with the ability to execute automatic concept tagging via both user-provided lexicon and string-pattern matching algorithm [23], this tool well fits the requirements of our annotation project and was chosen.

Although full-text articles could possibly provide more training materials and context information for NER models to learn from, retrieval issues such as free open access limited our project to using abstracts for corpus construction. In the future, for ‘open’ full-text articles under the Creative Commons License Agreement, one can take advantage of our ABAG-NER model or other NLP tools such as TeamTat, SpaCy, NLTK, some of which also have pre-trained models, to systematically annotate the literature of varied lengths (abstract or full-text). After constructing a corpus of both full texts and abstracts, some parameters of the deep learning model need to be modified or fine-tuned accordingly to adjust for the larger text capacity. Nevertheless, it is noteworthy that training models with full text articles do not always guarantee better model performance.

TeamTat (https://www.teamtat.org/) is a useful tool to semi-automatically annotate documents of any length, including full text journal articles and their figure legends [24]. TeamTat is a web-based concept tagging tool that is highly similar to ezTag. One advanced feature is that besides entity, TeamTat also allows annotation of relations, which is useful for future relationship extraction (RE) studies. Another highlight of TeamTat is that it facilitates team collaboration where multiple annotators can simultaneously but independently annotate or edit the same article [24]. This feature also results in inter-annotator agreement statistics for consistency assessment of the corpus, which was conducted in our study (Table 1). Despite the outstanding features, due to unknown technical issues with annotated output retrieval, we could not use TeamTat for the main annotation procedure and proceeded with ezTag instead.

With ezTag, we performed semi-automatic annotation for a balance of efficiency and quality. To elaborate, fully manual annotation would require a vast amount of specialized knowledge in immunology; experts would have to read through and annotate all abstracts from scratch, which is consuming both time and human resources. On the other hand, due to the ambiguity of natural languages, fully automatic annotation based on string-match algorithms was too rigid and erroneous. Noteworthy errors included conflicting tags, abbreviations or measurements mistaken as named entities, and polysemous or out-of-lexicon named entities not getting tagged. It is also possible to develop a more automatic pipeline in the future, with the advances of various NLP toolkits to be incorporated for systematic corrections of common errors. Nonetheless, to construct a corpus of the highest quality possible, manual correction is a must to account for cases that most programming scripts and tools would miss out. With semi-automatic annotation, the automatic string-matching step highlighted relevant mentions, so annotators knew where to focus right from the beginning of the manual annotation task; thus, they just needed to read the surrounding context to determine if the mentions were true tags. In brief, our procedure of semi-automatic annotation was substantially timesaving and did not require as much expert knowledge. More importantly, our ABAG corpus maintained its high quality and consistency with the IAA in the ‘almost perfect agreement’ score range.

To the best of our knowledge at the time of our study, there has been no corpus on antibody and antigen, which explains the need for this corpus construction. Nevertheless, since the majority of antigens are proteins, some are chemicals or nucleic acids, while some antibodies are also therapeutic drugs, our ABAG corpus actually shared noteworthy similarities with corpora on protein-gene (JNLPBA [20], ProGene [22]), on nanomedicines, and on pharmacogenomics (PGxCorpus [19], DDI [30]), etc. Regarding the JNLPBA corpus [20], F1 scores were 72.62% for BiLSTM-CRF [31], 73.50% for fusion-based Att-BiLSTM-CRF [31], 74.29% for BiLSTM-CRF with ELMo [32], 78.58% for CollaboNet [33], which is comprised of multiple BiLSTM-CRF models, and 77.59% for BioBERT [14]. For ProGene [22], the overall BioBERT F1 was 80.5%, which was 0.8% higher than our BioBERT result; each entity F1 ranged from 35.6 to 84.6%, while our highest was 87.2% for the antibody entity. Noticeably, BiLSTM-CRF based models and BioBERT, the two architectures studied in our project, achieved competitive NER performances on these protein-gene corpora.

In comparison with the related corpora, our results of both NER models (62.49% for BiLSTM-CRF and 81.44% for BioBERT) were decent for new entities that have not been studied before. The models are potential for further application and development, especially as novel entities: antibody and antigen. Besides, the ABAG-NER model was trained in the context of some molecule targeting, binding, or neutralizing some other molecule, so it can also be best expanded into text mining for other similar interactions, such as drug-target. To expand our pipeline for other targets in future studies, our ABAG-NER model can be used for the automatic annotation step, facilitating new corpora construction; the model is then retrained on the new corpus for the recognition of novel entities. Generalizing the recognized entities would be helpful for broader users.

For both models, NER performance for antibody was better than that for antigen, which could be owing to that the boundary for AB identification is more structured; there are typically more hinted words like “mAb” or “antibody” before or after AB mentions, creating clearer context around ABs that makes them easier to be recognized.

When analyzing the error cases on corpus test set as well as 8 out-of-corpus biomedical texts about antibody-antigen, major errors were generalized into three main causes:

### Tagging inconsistency problem

**Fig. 5:**
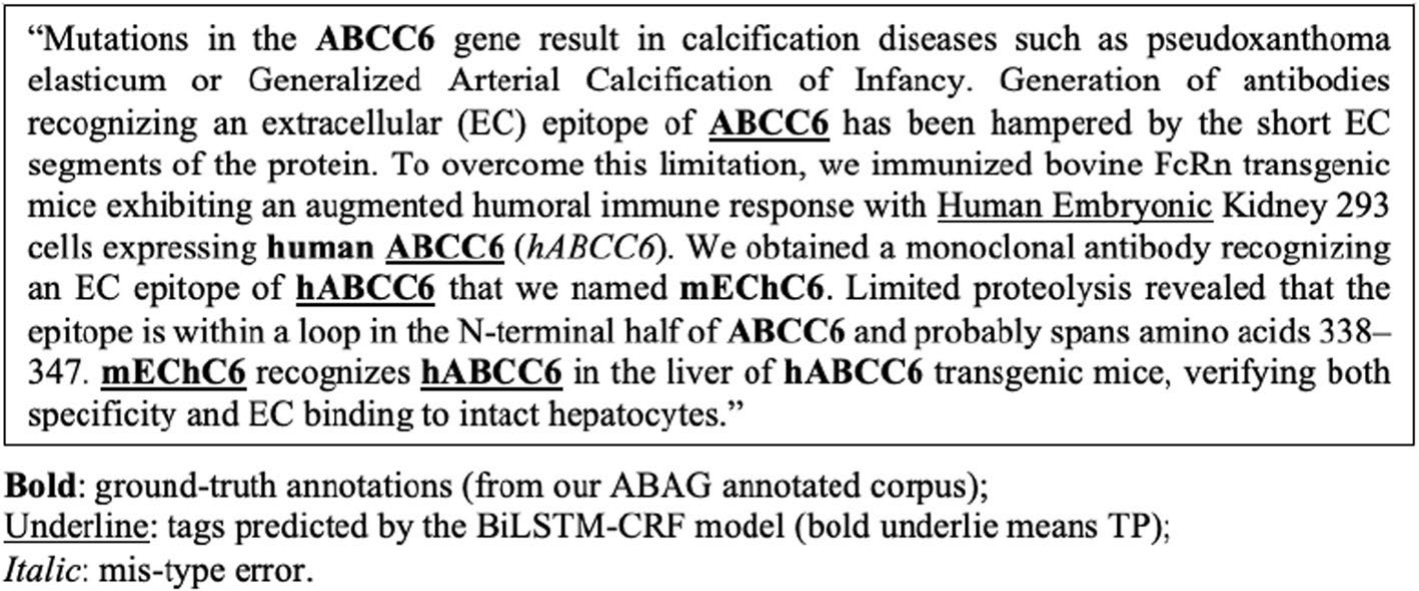
An example of tagging errors by the BiLSTM-CRF model in comparison with the ground truth

Let consider an abstract sample with common error cases shown in Fig. 5.

We can see from Fig. 5 that the main error type, in this case, was FNs. The 2 antigen names: ABCC6 and hABCC6 (abbreviation of human ABCC6), and 1 antibody name “mEChC6” appeared several times, but half of the times their mentions were not tagged by the BiLSTM-CRF model. This is called tagging inconsistency problem. In this abstract, the mentions with surrounding keywords like “epitope of” or “recognizes” were tagged, while ones further away were not, probably due to fade of context clues. This problem caused a low recall rate.

On the contrary, the BioBERT model achieved relatively high recall scores; error analysis also validated that BioBERT mitigated the tagging inconsistency problem faced by BiLSTM-CRF, thus producing better coverage of name mentions (BioBERT model correctly recognized all ABAG names in the abstract in Fig. 5).

### Span mismatch

Span mismatch is when the tag predicted by a NER model has overlapping text but does not exactly match with the ground-truth annotation. An example of span mismatch can be seen in the above example (Fig. 5), where the model recognized “ABCC6” instead of “human ABCC6”. As indicated in our annotation guidelines, we decided to include extra important information for the ABAG such as species, year, location, strain, etc. along with ABAG names as one long mention. Nevertheless, the NER model sometimes failed to tag this information along with the entity names, possibly due to lack of such occasions/examples in the corpus to learn from.

**Fig. 6:**
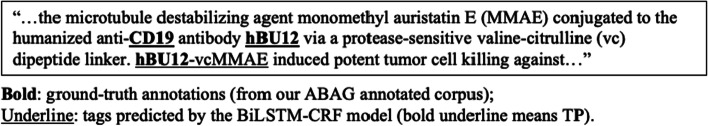
An example of span mismatch by the BiLSTM-CRF model in comparison with the ground truth

On the contrary, in cases of antibody-drug conjugate (ADC), such as “hBU12-vcMMAE” (in the example in Fig. 6), where hBU12 is an AB and vcMMAE is the conjugated drug linker, we decided to annotate only the antibody part, which is “hBU12” instead of “hBU12-vcMMAE”. Unfortunately, many ADCs have their whole name formats similar to a typical antibody name, which confuses the model to annotate both parts like “hBU12-vcMMAE” as a whole tag (as illustrated in Fig. 6), leading to span-mismatch error.

Another major source of confusion comes from elliptical coordinated compound noun phrases with special characters (e.g., “ + ”, “−”, “/”, “.” and brackets), and/or conjunction words (“and”, “or”) in between their names. For example, “interleukin (IL)-12”, “IgG 24 and 30”, or “TGF-beta1, 2, and 3” are such confusing AG names, of which annotation could be several tags separated by the conjunctions or just one single tag of the whole phrase.

However, these span mismatch errors actually have a minor effect on the meaning conveyed by the annotations; the basic information of ABAG is still delivered. For this reason, our report of relax-match scoring accounted for this slight difference, or also known as soft agreement. In span-relax match, the NER F1 increased by over 10% to achieve 71.7% for the BiLSTM-CRF model (Table 5) and increased by about 7% to achieve 87.92% for the BioBERT model (Table 6).

### Corpus annotation inconsistency

**Fig. 7:**
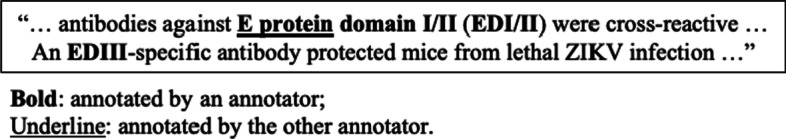
An example of corpus annotation inconsistency between two annotators

Despite high IAA, there existed annotation inconsistency in our corpus for vastly ambiguous cases. An example is demonstrated in Fig. 7.

This excerpt is from a doubly annotated abstract. The two annotators agreed to tag “E protein” as AG but disagreed on the domain name. A rigid rule could not be established to decide the annotation for such names or cases as they differ based on their contexts. For AB, the main confusion lies in their name formats since there’s no specific or consistent rule for naming antibody in published literature; many names are just numbers or abbreviations that are easily confused with measurements, cell lines, or gene mutations. For AG, some names of target domain, site, or virus may be AGs in some context but may be considered either too specific or not specific enough to be tagged as AGs in other contexts. This was why annotators faced inconsistency problems during the manual annotation task, which unfortunately caused confusion in the learning of NER models. As expected, inconsistent annotations occurred mostly at the beginning of the annotation process. As we proceeded, confusing cases (over 100 abstracts in total) were discussed weekly and agreeably solved together by all three annotators. Through this process, potential sources that might lead to disagreement were also realized and the annotation guideline was updated accordingly to improve the stringency of the tagging rules for higher consistency. Ultimately, the Inter-Annotator Agreement score ranging from 91.46 to 94.31% implied that inconsistently annotated cases accounted for a small percent of the final corpus. For future studies, provided time and human resource availability, we suggest revising the corpus for another one or more rounds of annotation with our latest set of rules (most stringent annotation guideline).

In our study, there were no situations where the BiLSTM-CRF performed better. The BioBERT-based model outperformed the BiLSTM-CRF-based model (with approximately 20% higher in F1 score for both ABAG entities). However, in some other situations [34], LSTM could perform better than BERT. Noticeably, in our ABAG-NER study, BioBERT had better coverage (or higher sensitivity), which was best explained by the fact that it mitigated the tagging inconsistency problem faced by BiLSTM-CRF. In other words, if an AB/AG name appears multiple times in an abstract, BioBERT would likely recognize the instances where BiLSTM-CRF might miss. The distinction can best be attributed to three main reasons. First, the attention mechanism of a transformer advances BioBERT in handling long-term dependencies for consistent labeling across multiple occurrences of the same entity [35]. Second, BioBERT was pre-trained on two large biomedical corpora for better biomedicine-specific word representations [14]. Third, the word-piece tokenization used in BERT has advantages in recognizing unfamiliar words (novel entities) by splitting them into smaller known tokens and providing meaningfully context-specific representation for each [35]. In brief, the BioBERT-based model is recommended for ABAG-NER tasks.

With its promising performance, the BioBERT model (https://github.com/TrangDinh44/ABAG_BioBERT.git) can be further developed into a text mining tool. As emergent databases are providing more relevant antibody-antigen articles, one can use this tool to constantly and automatically annotate and extract AB/AG names from these newly published articles. The extracted information and annotated articles can serve 2 purposes. First, the recognized AB/AG names from these articles (if novel) can be used to update many relevant databases, where users can look up potential cross-reacted targets of a novel AB or search for new ABs targeting a certain AG. Second, the automatically annotated articles can be revised manually and added onto our current corpus. With high-quality ABAG-NER performance (F1 = 81.44%), the manual editing should be much faster and easier with less erroneous tags. This expanded corpus can be used to retrain the ABAG-NER models. Theoretically, increasing corpus size with more diverse examples is beneficial for the model to learn from, which would ultimately enhance its performance. This strategy works as an interactive learning cycle.

To demonstrate a specific use case for the tool, we queried PubMed with the keyword “monoclonal antibody therapy” for this topic is rapidly growing in terms of demand and application. In 2022 only, 9,518 articles with available abstracts were published. After performing NER on these abstracts, a total of 27,603 AB/AG mentions were recognized. This is a quick and automated process to extract the latest data to update databases about monoclonal antibodies or therapeutic antibodies. A specific result for the AG “Omicron” demonstrated that Omicron was co-mentioned with 60 different antibodies across 36 abstracts, including Sotrovimab (14 abstracts), Casirivimab (8 abstracts), and Imdevimab (7 abstracts) as the topmost frequent co-mentions (Table 7). Besides the well-studied antibodies, results also included novel compounds of high therapeutic potential from latest preclinical data (e.g., IMM-BCP-01 antibody cocktail) or computational discoveries (e.g., AKS01-05 against the Spike protein of Omicron). Supporting abstracts are annotated, and potentially ranked by ABAG name relevancy, frequency, or users’ feedback, so users can pinpoint a list of relevant studies regarding anti-Omicron antibodies they can explore more or follow up.

**Table 7:** A use case study demonstrating NER-extracted antibodies against Omicron from relevant abstracts

Antibodies	Number of supporting abstracts
SOTROVIMAB	14
CASIRIVIMAB	8
IMDEVIMAB	7
TIXAGEVIMAB	4
CILGAVIMAB	4
ETESEVIMAB	3
AZD1061	3
BAMLANIVIMAB	3
BEBTELOVIMAB	3
EVUSHELD	3
CT-P59	2
VIR-7831	2
S2K146	2
BNT162B2	2
REGDANVIMAB	1
IMDEVIMA	1
CASIVIRIMAB	1
AZD8895	1
COV2-2196	1
COV2-2130	1
LY-COV016	1
LY-COV555	1
REGN10987	1
REGENERON	1
REGN10933	1
S309	1
RONAPREVE	1
ADINTREVIMAB	1
S2H97	1
S2X259	1
ZF2001	1
S304	1
AKS-05	1
AKS-03	1
AKS-01	1
AKS-04	1
AKS-02	1
OCRELIZUMAB	1
P5C3	1
P2G3	1
ZWD12	1
P2B-2F6	1
CB6	1
REGN	1
B38	1
P2B2F6	1
CR3022	1
BRII-196	1
DXP-604	1
ADG20	1
SI-F019	1
IB20	1
IB14	1
58G6	1
IMM20253	1
IMM20190	1
IMM-BCP-01	1
IMM20184	1
35B5	1
ZCB11	1

Sixty different antibodies were co-mentioned with “Omicron” in 36 abstracts. The antibodies were arranged by the number of abstracts they appeared in (in descending order).

Lately, a rising fusion architecture of Att-BiLSTM-CRF, introduced by Luo et al. in 2018, which leverages an attention mechanism to pay special attention to a similar entity mentioned multiple times throughout the whole document, has been shown to effectively alleviate this tagging inconsistency problem [36]. Suggestion for future studies is to combine the attention layer with our BiLSTM-CRF for sufficient capture of multi-mentioned entity names and a significant decline in FN errors. Future research can also focus on model improvement by combining BiLSTM-CRF with SOTA pre-trained biomedical word-embedding models (such as BioBERT). Besides, further development for corpus can include annotating AB-AG relations and/or more specific entities, like types of antibodies, nanobody, paratope and epitope, etc.

## Conclusions

We established and annotated an Antibody-Antigen corpus consisting of 3210 abstracts. With this corpus, we developed and optimized two baseline models—BiLSTM-CRF and BioBERT—specialized for NER tasks on AB and AG domains. F1 scores of the models are 62.49% and 81.44%, respectively, which demonstrated potential for further development on the two novel entities and possibly their relation. The application of these SOTA models in building an ABAG-NER tool kit would help users automatically extract central information about ABs and AGs from biomedical literature.

## Data Availability

Programming language: Python. The ABAG-annotated corpus generated and analyzed during the current study is available on https://bit.ly/CBB_ABAG_corpus. The NER model generated and analyzed during the current study is available in the Github repository: https://github.com/TrangDinh44/ABAG_BioBERT.git.

## Abbreviations

AB: Antibody (as a named entity)
ABAG: Antibody and/or antigen (as named entities)
ABCD: Antibodies chemically defined
ADC: Antibody-drug conjugate
AG: Antigen (as a named entity)
Att-BiLSTM-CRF: Attention-based BiLSTM-CRF
BERT: Bidirectional encoder representations from transformers
Bi-LSTM: Bidirectional long-short term memory
BioNER: Biomedical named entity recognition
BioNLP: Biomedical natural language processing
BioBERT: Biomedical bidirectional encoder representations from transformers
CNN: Convolutional neural network
CRF: Conditional random fields
FN: False negative
FP: False positive
IAA: Inter-annotator agreement
IOB: Inside, outside, begin
NCBI: National Center for Biotechnology Information
NER: Named entity recognition
NLP: Natural language processing
LSTM: Long-short term memory
P: Precision
PMID: PubMed identifier
R: Recall
RAM: Random access memory
RNN: Recurrent neural network
SOTA: State-of-the-art
TP: True positive
XML: Extensible markup language
